# Meiotic Crossover Patterning

**DOI:** 10.3389/fcell.2021.681123

**Published:** 2021-07-22

**Authors:** Nila M. Pazhayam, Carolyn A. Turcotte, Jeff Sekelsky

**Affiliations:** ^1^Curriculum in Genetics and Molecular Biology, The University of North Carolina at Chapel Hill, Chapel Hill, NC, United States; ^2^Department of Biology, The University of North Carolina at Chapel Hill, Chapel Hill, NC, United States; ^3^Integrative Program for Biological and Genome Sciences, The University of North Carolina at Chapel Hill, Chapel Hill, NC, United States

**Keywords:** meiosis, recombination, interference, centromere, crossover assurance

## Abstract

Proper number and placement of meiotic crossovers is vital to chromosome segregation, with failures in normal crossover distribution often resulting in aneuploidy and infertility. Meiotic crossovers are formed via homologous repair of programmed double-strand breaks (DSBs). Although DSBs occur throughout the genome, crossover placement is intricately patterned, as observed first in early genetic studies by Muller and Sturtevant. Three types of patterning events have been identified. Interference, first described by Sturtevant in 1915, is a phenomenon in which crossovers on the same chromosome do not occur near one another. Assurance, initially identified by Owen in 1949, describes the phenomenon in which a minimum of one crossover is formed per chromosome pair. Suppression, first observed by Beadle in 1932, dictates that crossovers do not occur in regions surrounding the centromere and telomeres. The mechanisms behind crossover patterning remain largely unknown, and key players appear to act at all scales, from the DNA level to inter-chromosome interactions. There is also considerable overlap between the known players that drive each patterning phenomenon. In this review we discuss the history of studies of crossover patterning, developments in methods used in the field, and our current understanding of the interplay between patterning phenomena.

## Meiotic Recombination

Crossovers are generally avoided during repair of DNA double-strand breaks (DSBs) in mitotically proliferating cells, presumably because they can lead to loss of heterozygosity or to chromosome rearrangement (when occurring between non-allelic repetitive sequences) (reviewed in Andersen and Sekelsky, 2010). To avoid crossovers, non-crossover outcomes are promoted through the actions of helicases that disassemble recombination intermediates (reviewed in Huselid and Bunting, 2020). In contrast, in meiotic cells DSBs are induced enzymatically and repaired to ensure that some become crossovers, which lead to chiasmata that promote accurate segregation of homologous chromosomes (Hawley, 1988). This change in outcome is achieved through the addition of numerous meiosis-specific elaborations to DSB repair.

**FIGURE 1:**
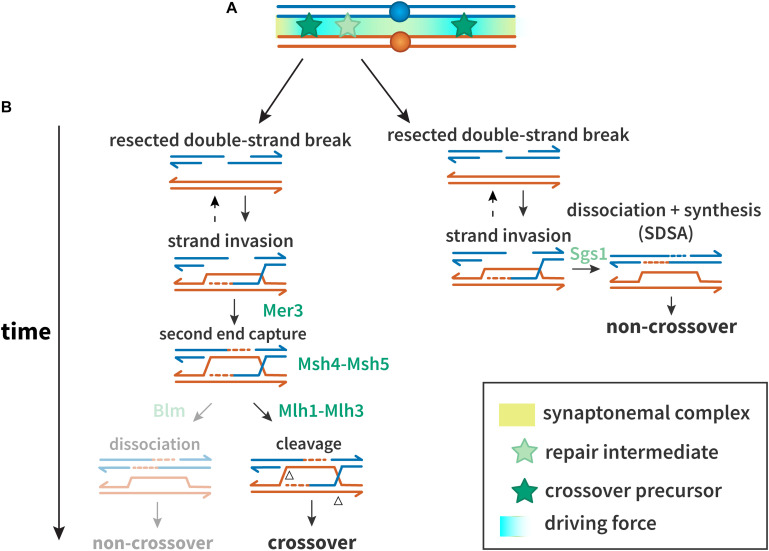
Proteins and mechanisms of crossover interference. **(A)** A driving force (teal), potentially protein aggregation or mechanical stress, results in designation of crossover precursors (stars, dark green) at spaced intervals. Repair intermediates that do not experience sufficient driving force (stars, light green) are not so designated, and become non-crossovers. **(B)** Interference at the DNA level. Crossover precursors are processed through the class I crossover pathway, involving the ZMM proteins. Mer3 promotes second end capture, and Msh4–Msh5 clamp to and stabilize branched molecules to prevent their dissociation and to recruit Msh1–3. Intermediates are resolved by Mlh1–Mlh3 to generate crossovers. Non-crossovers in yeast are thought to be exclusively processed through the synthesis-dependent strand annealing (SDSA) pathway, promoted by Sgs1. In other organisms, the Sgs1 ortholog Blm may also promote formation of non-crossovers after second end capture.

The first step in homology-directed repair of DSBs is resection of the DSB ends (Figure 1) (reviewed in Heyer et al., 2010). In miotic cells, the recombinase Rad51 promotes strand exchange with a homologous duplex that is used as a template for synthesis; in meiosis, the meiosis-specific Rad51 paralog Dmc1 is used instead of or in conjunction with Rad51 (reviewed in Shinohara and Shinohara, 2004). As in mitotic DSB repair, dissociation of the nascent strand from the template allows completion of repair by synthesis-dependent strand annealing (SDSA), a pathway thought to generate most or all non-crossovers (Allers and Lichten, 2001; Marsolier-Kergoat et al., 2018). In the major meiotic crossover pathway, the template strand that is displaced by synthesis anneals to the other resected end of the broken chromatid, and further synthesis and ligation leads to a double-Holliday junction (dHJ) structure, which is resolved into crossover products (Figure 1).

A defining feature of the major meiotic crossover pathway is dependence on a group of proteins referred to as ZMM (Zip, Msh, and Mer). In *Saccharomyces cerevisiae*, the ZMMs consist of at least seven players: Zip1, Zip2, Zip3, Zip4/Spo22, Spo16, Mer3, Msh4, and Msh5. Zip1 is a structural component of the synaptonemal complex (SC), a proteinaceous structure that assembles between homologous chromosomes during prophase I (Sym et al., 1993). Msh4 and Msh5, homologs of the *Escherichia coli* mismatch repair protein MutS, form a heterodimer termed MutSγ, which is thought to promote crossing over by stabilizing pre-crossover intermediates to block SDSA and/or promote formation of dHJs (Snowden et al., 2004; Jessop et al., 2006; Oh et al., 2007).

Recent studies have identified an important role for proteolysis of ZMM proteins in promoting crossovers in meiosis. Ahuja et al. (2017) found that the proteasome is necessary for chromosomes to pair and for crossover-designated DSBs to effectively be repaired as crossovers. The proteasome is recruited to chromosomes by Zip1 and Zip3. SUMO modification mediated by the meiotic E3 ligases RNF212 and HEI10 is thought to act like a checkpoint in mouse, pausing recombination by inducing degradation of various recombination factors (Rao et al., 2017). Likewise, Msh4 has been shown to be targeted by a degron for ubiquitin-independent proteolysis (He et al., 2020). This degron is under control of the kinase Cdc7, and phosphorylation of the degron permits Msh4–Msh5 to promote crossovers.

The final steps in crossover formation are also modified in meiosis. In mitotic cells, it is believed that the primary pathway for processing dHJs involves unwinding Blm helicase and decatenation by Topoisomerase 3α, a process that generates only non-crossovers (Plank et al., 2006). An alternative is resolution of the Holliday junctions by structure-selective endonucleases, which can produce crossover or non-crossover products (Wyatt and West, 2014). In meiosis, however, most or all dHJs are processed to generate crossovers (Allers and Lichten, 2001), through the action of a complex containing MutLγ, a heterodimer of Mlh1 and Mlh3, and other proteins (Zakharyevich et al., 2012; Cannavo et al., 2020; Kulkarni et al., 2020).

**FIGURE 2:**
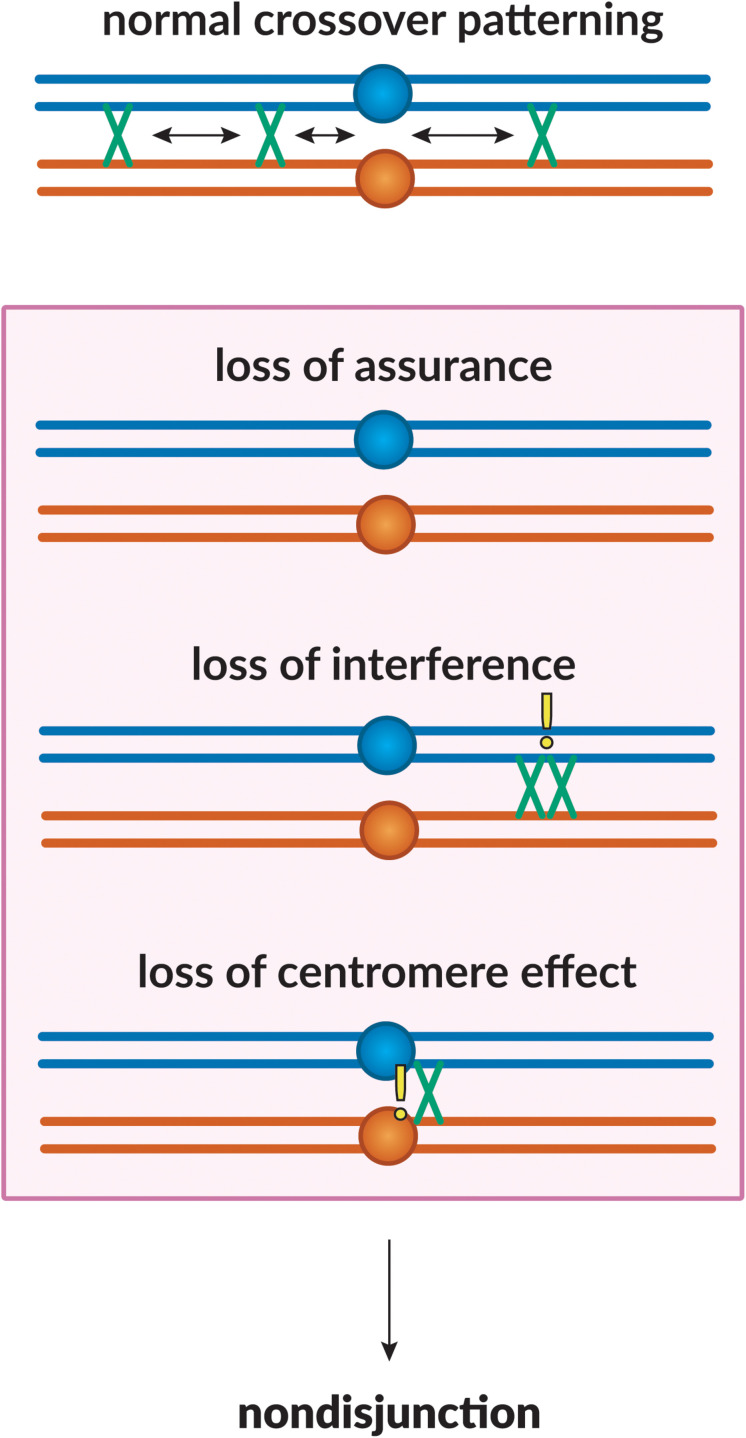
Crossover patterning phenomena. The proper placement of crossovers along the chromosome is governed by three patterning phenomena. Homologous chromosomes are shown in blue and orange, with crossovers between them shown in green. Loss of assurance results in a lack of crossing-over between a pair of homologs; loss of interference results in two crossovers being placed in close proximity to one another; loss of the centromere effect results in centromere-proximal crossovers. These phenomena can lead to a failure in proper chromosome segregation, leading to non-disjunction.

The meiotic DSB repair process outlined above ensures that some DSBs will be repaired as crossovers. However, the determination of which become crossovers and which are repaired as non-crossovers is highly regulated to ensure that all chromosome pairs receive at least one crossover, that crossovers are in positions that facilitate segregation, and that crossovers are excluded from regions where they might impede segregation (Koehler et al., 1996b). The phenomena that achieve this are collectively referred to as “crossover patterning.” Below, we discuss various meiotic crossover patterning phenomena – interference, assurance, and suppression (Figure 2), from initial recognition to current understanding.

## Interference

### History

Crossover interference was originally observed by Sturtevant when constructing the first linkage map in *Drosophila melanogaster* in 1913 (Sturtevant, 1913). His observation that one crossover makes the occurrence of another less likely was based on the results of counting double crossovers (DCOs) between six sex-linked factors, where he observed that DCOs between two adjacent intervals were much lower in frequency than should be expected by chance. This phenomenon, termed interference by Muller, was later shown by Sturtevant to be an intra-chromosomal process (Sturtevant, 1915).

Muller later showed that interference is limited to crossovers on the same chromosome, with a crossover on one chromosome having no effect on crossing over on another (Muller, 1916a). Muller further noted, just as Sturtevant had, that the reach of interference seemed to depend on the distance of the intervals being considered, with longer intervals showing less interference than shorter ones. Weinstein (Weinstein, 1918) confirmed this by showing that interference decreases with distances up to 46 map units (a measure of recombination in *D. melanogaster* equivalent to centiMorgans) from the initial crossover.

**FIGURE 3:**
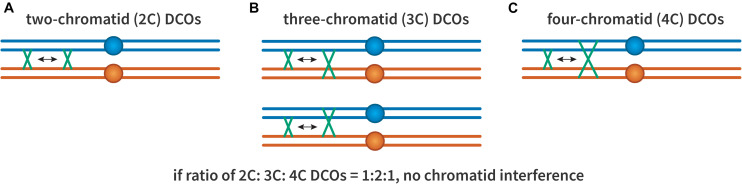
Chromatid interference. Each panel illustrates two crossovers on one bivalent, with each line representing one double-stranded DNA chromatid. The first (leftmost) crossover is the same in all cases, occurring between the two inner chromatids. **(A)** A 2-chromatid DCO. **(B)** Two possible 3-chromatid DCO configurations. **(C)** A 4-chromatid DCO. If there is no chromatid interference, the ratio of 2:3:4 should be 1:2:1.

The possibility of chromatid interference was also considered (Figure 3): When there are two crossovers on the same chromosome arm, do the chromatids involved in one affect which are used in the other? This question was first investigated by Emerson and Beadle (1933). They found that in *Drosophila* the first crossover did not seem to influence which chromatids were involved in the second, suggesting no chromatid interference. The same result was also shown in both budding and fission yeast (Mortimer and Fogel, 1974; Munz, 1994), though not in *Neurospora crassa* (Perkins, 1962). Nonetheless, most studies have assumed no chromatid interference (Mather, 1935; Chakraborty et al., 2017).

### Interfering and Non-interfering Crossovers

Not all crossovers participate in interference. Based on studies of recombination mutants in *Caenorhabditis elegans*, Zalevsky et al. (1999) proposed the existence of two meiotic recombination pathways. In most organisms, the primary meiotic crossover pathway (described above and in Figure 1) produces crossovers that are subject to interference, while other pathways produce crossovers that are indifferent to interference (de los Santos et al., 2003; Argueso et al., 2004; Börner et al., 2004; Higgins et al., 2004; de Boer et al., 2006; Holloway et al., 2008). This results in some crossovers participating in interference and others neither contributing to nor responding to interference. These have been called classes I and II crossovers, respectively.

Class II crossovers were initially identified as being generated independently of MutSγ (Zalevsky et al., 1999; de los Santos et al., 2003), but the class II designation has been used in different ways. For example, when a blockage occurs in the primary pathway due to a missing component, backup mechanisms will complete repair (De Muyt et al., 2012; Zakharyevich et al., 2012; Kohl and Sekelsky, 2013). These backup pathways, like mitotic DSB repair, are not directed toward a crossover outcome. Nonetheless, crossovers do occur in some instances, but this requires structure-selective nucleases such as Mus81–Mms4 rather than MutLγ (de los Santos et al., 2003; Argueso et al., 2004; Berchowitz et al., 2007; Holloway et al., 2008). Some consider these to be class II crossovers because they come from a different pathway, but if the blockage happens after crossover designation has occurred and interference has been enforced, these crossovers may exhibit interference.

The proteins involved and percentage of crossovers attributed to classes I and II differ among organisms. In budding yeast, only about 60% of meiotic crossovers come from the class I pathway (de los Santos et al., 2003; Medhi et al., 2016). Unique features of this pathway are similar in *Arabidopsis* and mouse, with ZMM proteins and MutLγ playing central roles (Falque et al., 2007; Higgins et al., 2008; Lu et al., 2008; Svetlanov et al., 2008); however, class I crossovers comprise 75–90% of all meiotic crossovers in *Arabidopsis* (Berchowitz et al., 2007; Higgins et al., 2008) and more than 90% of crossovers in mouse meiosis (Baker et al., 1996; Holloway et al., 2008).

In *Drosophila*, 90–100% of crossovers come from a class I pathway (Baker and Carpenter, 1972), but Dmc1, Msh4, and Msh5 are absent from fly genomes (Sekelsky, 2017). Kohl et al. (2012) hypothesized that crossover-designated recombination intermediates are instead stabilized by the MCM-like proteins Mei-217, Mei-218 and Rec. Furthermore, the class I resolvase function of Mlh1–Mlh3 is replaced by the XPF ortholog Mei-9 and its partners Ercc1 and the Slx4 ortholog Mus312 (Sekelsky et al., 1995; Yıldız et al., 2002; Radford et al., 2005). All crossovers in *C. elegans* are processed through the ZMM-based class I pathway (Zalevsky et al., 1999), though it also uses resolvases other than MutLγ (Saito et al., 2009; Agostinho et al., 2013; O’Neil et al., 2013). *Schizosaccharomyces pombe* appears to lack most of the specialized features of the class I pathway, including ZMM proteins, and most crossovers are dependent on Mus81 and exhibit little or no interference (Smith et al., 2003; Fowler et al., 2018).

### Measures of Interference

Interference can be measured based on genetic distance (recombination frequencies between genetic loci) or physical distance (chromosome axis length or distance in base-pairs between crossovers from whole-genome sequencing). The main methods in use are described below.

#### Coefficient of Coincidence

Although development of the genetic map by Sturtevant (1913, 1915) and Bridges (1915) was integral to understanding interference, a better measure to directly measure interference was soon developed. In 1916, Muller defined coincidence as the occurrence of a crossover in each of two adjacent intervals (i.e., a double-crossover, DCO) (Muller, 1916b). Muller measured strength of interference through the ratio between the number of observed DCOs and the number of DCOs expected if the two intervals are independent (no interference). This measure is now referred to as the coefficient of coincidence (*CoC*). Interference (*I*) is represented as 1 - *CoC*. If *CoC* is one, then *I* is zero and there is no interference between the two intervals in question; if *CoC* is between zero and one there is interference between the two intervals (negative interference is also possible but not considered here). This method is often used to calculate strength of interference based on counts of crossovers in specific intervals, as in genetic studies.

#### Interference in Tetrads

The use of fungi as genetic model organisms allows recovery of all products of meiosis in tetrads or octads, making it possible to calculate interference with just two markers. If a strain that is heterozygous for two linked markers, say *A B* on one homolog and *a b* on the other, goes through meiosis to produce a tetrad with four spores, three outcomes are possible. Parental ditype (two *A B* spores and two *a b* spores) results when there has not been a crossover between the two loci or a 2-strand DCO (Figure 3). Non-parental ditype (two *A b* spores and two *a B* spores) results from a 4-strand DCO, and tetratype (*A B, A b, a B, a b*) results from a single crossover or a 3-strand DCO. Papazian (1952) developed a simple equation to calculate interference from the observed numbers of each class. Although widely used, these formulas are applicable only to datasets within certain parameters, and determining statistical significance between datasets if difficult. Stahl (2008) published a method termed “The Better Way,” that overcomes these limitations.

#### Cytological Measures of Interference

Chiasma interference has been measured cytologically in humans and other organisms. Hulten (1974) measured the length of each chromosome and counted chiasmata in chromosome spreads of a human testicular sample. Hulten (1974) mapped the distribution of chiasmata on chromosome arms and determined interference based on the mean crossover count compared to the variance of the data. Jones (1984) noted that this measurement is flawed in that it does not consider the position of crossovers along the chromosome. He expanded on this approach by arbitrarily dividing the chromosome arms into even intervals to calculate *CoC* using chiasmata from cytological data. Hulten later developed the Chiasma Interference Map (CHIM), in which chiasmata are marked along the chromosome and chiasmata that occurred on the same chromosome are joined by a loop. The strength of interference can be visualized via the size and quantity of the loops relative to the total number of chiasmata, with longer loops indicating stronger interference (Hulten, 2011).

Fluorescence microscopy allows measurement of interference based on other cytological markers. MLH1 marks sites of crossovers in mammalian meiosis, and distances between foci along a bivalent can be used to estimate interference (Froenicke et al., 2002). Zip3 has been used as a similar marker in budding yeast (Zhang et al., 2014b). These measurements of interference are unique in counting only class I crossovers, whereas other methods include both class I and class II crossovers in calculations of interference. It is also notable that these methods mark crossover sites during pachytene, while chiasmata are apparent later, during diakinesis and metaphase I.

#### Measuring Interference Using Whole-Genome Sequencing

In whole-genome genotyping or sequencing, the markers (e.g., single nucleotide polymorphisms) are so dense that each interval is too small to have enough crossovers for *CoC* analysis. One method to circumvent this problem is to divide the chromosome into arbitrary intervals of equal length and use these to calculate *CoC* or to fit a gamma distribution (Broman et al., 2002; Anderson et al., 2015). Another is to compare the average distance between crossovers on chromosomes with multiple crossovers (Miller et al., 2016).

#### Modeling Interference Data

A more recent way to compare interference between datasets is to make use of computational models that simulate interference, such as the beam-film model (discussed below) (White et al., 2017). The beam-film program can fit experimental data to a model and contains a parameter (*L*) that reflects the strength of interference.

It should be noted that these methods differ in profound ways. Chromosome length is measured in recombination frequency in the genetic methods, in meiotic chromosome axis length in cytological methods, and in DNA sequence length in sequence-based methods. Also, counting of MLH1 or Zip1 foci includes only class I crossovers, but other methods include both class I and class II crossovers. The beam-film model can accommodate date from genetic, cytological, or sequence studies, and has a parameter to include class II crossovers.

### Factors Influencing Interference

#### Temperature

The influence of temperature on crossover frequencies was first studied by Plough, Stern, and Graubard in the early 20th century (Plough, 1917a,b; Stern, 1926; Graubard, 1934). Schweitzer (1935) analyzed Graubard’s data and concluded that temperature does not affect interference in *Drosophila*, and that the average distance between two crossovers does not change at different temperatures. However, a more recent study in *A. thaliana* showed that increased temperatures lead to an increase in overall crossover frequencies through the formation of additional class I crossovers, suggesting that physical measures of interference are decreasing (De Storme and Geelen, 2020).

#### Chromosome Size

Small chromosomes exhibit higher rates of crossing over per physical unit than larger chromosomes (Kaback et al., 1989; Mortimer et al., 1989; Riles et al., 1993), and the smallest chromosome in *S. cerevisiae* shows increased recombination when split into two smaller segments (Link and Olson, 1991). Kaback et al. (1999) observed that interference increased with the size of the chromosome. Intervals of the same cytological length showed greater interference when relocated to larger chromosomes, leading the authors to conclude that the size dependency of meiotic recombination rates is mediated at least in part by crossover interference. Kaback et al. (1999) hypothesized that the recombination machinery initiates crossover formation on larger chromosomes earlier than on smaller ones because they are larger targets. This leads to an interference signal “spreading” along the chromosome on either side of the crossover, rendering many areas of the larger chromosomes unable to form another crossover and leading to smaller chromosomes having less competition for the recombination machinery. The authors proposed that when interference begins to act on larger chromosomes there is more of some rate-limiting component available per unit of recombination-proficient DNA. This results in the remaining rate-limiting component promoting crossing over at greater rates on smaller chromosomes. Thus, the size dependency of recombination rates is explained by larger chromosomes initiating crossing over earlier and having longer interference tracts. Many rate-limiting factors in the recombination machinery have been identified (Reynolds et al., 2013; Qiao et al., 2014; Ziolkowski et al., 2017; He et al., 2020).

In contradiction to this hypothesis, Stahl et al. (2004) showed that shorter chromosomes had more class II crossovers than longer chromosomes, suggesting that the chromosomal size dependence of recombination rates isn’t due to changes in the strength of interference, but is instead due to a difference in the proportion of class I versus class II crossovers.

More recently, Murakami et al. (2020) have shown that in short chromosomes of budding yeast there is a greater recruitment of DSB proteins, indicating a higher density of DSBs. Their data also showed that shorter chromosomes tend to stay in a DSB-competent state for longer; however, no inferences can be made about how this may affect the process of interference in these chromosomes, as it is possible, based on the study by Stahl et al. (2004) that the extra DSBs are being repaired as class II crossovers.

#### Synaptonemal Complex

The synaptonemal complex (SC) was first suggested to be important for crossover interference when it was reported that *Aspergillus nidulans* and *S. pombe*, two organisms that do not have an SC, also do not exhibit interference (Moens, 1969; Snow, 1979; Egel-Mitani et al., 1982; Bähler et al., 1993; Munz, 1994). Concomitant defects in interference and synapsis were also seen in *as1* and *asb* mutants in tomato (Moens, 1969; Havekes et al., 1994). Further, organisms heterozygous for chromosomal translocations do not display interference in the region of the rearrangement, where SC continuity is presumably disrupted (Arana et al., 1987; Parker, 1987).

Studies in budding yeast suggest that the relationship between SC and interference is more complex. Zip1 is a component of the SC, and *zip1* mutants fail to build SC (Sym et al., 1993). Based on tetrad analysis in *zip1* mutants, Sym and Roeder (Sym and Roeder, 1994) concluded that interference requires the SC, and suggested that Zip1 could be the polymer that diffuses outward from crossover sites as in the model polymerization model proposed by King and Mortimer (see below). This requirement of Zip1 and its orthologs for crossover interference has also been shown by numerous other studies (Hayashi et al., 2010; Libuda et al., 2013; Duroc et al., 2014; Voelkel-Meiman et al., 2015; Rog et al., 2017; Capilla-Perez et al., 2021). However, it is important to note that since Zip1 mutants are defective in class I crossover formation, the apparent reduction in interference may be due to the remaining crossovers being class II. Chua and Roeder (1997) further supported the idea that the SC plays a role in interference when they showed that budding yeast mutants lacking the telomere-associated meiotic protein TAM1, also known as Ndj1 and required for telomeric clustering during prophase I as well as proper homolog pairing and disjunction (Conrad et al., 1997; Trelles-Sticken et al., 2000), had defects in both chromosome synapsis and crossover interference. They attributed this to TAM1 playing a role in homolog pairing, leading to defects in alignment and synapsis.

In contrast, Fung et al. (2004) observed that synapsis initiation complexes (SIC), identified as Zip2 foci, exhibit interference, suggesting that crossing over happens at these initiation sites, as had been suggested earlier by Egel (1978). Since SICs assemble prior to SC formation and are still present in a *zip1* mutant where SC is absent, Fung et al. (2004) concluded that interference in budding yeast can act independently of synapsis. This independence has also been shown in *Sordaria* (Zickler et al., 1992). A 2006 study showed substantial interference between MSH4 foci in mice with defective SCs, although the data did not allow determination of whether MLH1 foci interfere in the absence of the SC (de Boer et al., 2006). It is also important to note that *zip1* mutants often undergo delays in prophase (Sym et al., 1993), which may be true in the *tam1* mutant as well, suggesting that the SC’s apparent role in interference could be due to cell cycle defects. Thus, the role of the synaptonemal complex in interference remains enigmatic.

### Interference Models

Since the discovery of crossover interference, several models have been proposed to describe this phenomenon. We briefly describe the most influential models below, in chronological order; additional discussions can be found in Berchowitz and Copenhaver (2010) and Otto and Payseur (2019).

#### Polymerization Model

The polymerization model proposed by King and Mortimer (King and Mortimer, 1990) hypothesized that early recombination structures are randomly distributed across the chromosome, and that once crossing over is initiated at the positions where these structures attach, a polymerization reaction is triggered which would then inhibit crossovers in neighboring regions by preventing the binding of other early structures to the SC. This polymerization reaction extends bidirectionally from each crossover site, explaining why interference decreases with distance from the original crossover. King and Mortimer also put forth a computational simulation of the model that they were able to fit well to *Drosophila* and budding yeast crossover data. While no polymer has been identified that fits this model, it agrees well with an earlier interference model proposed by Egel (1978) suggesting that crossover interference is a result of synapsis. Maguire (1977) put forth the idea that establishment of crossover sites may occur before synapsis is initiated, and that while the SC may be important for the process of crossing over, its “deployment” along the chromosome may have another function. Based on her arguments, Egel argued that if crossover sites are formed before synapsis and are also points of nucleation for the SC, it is this formation and zippering of the SC that prevents crossovers in neighboring sites. According to this model, crossovers cannot form in chromosomal regions that have already synapsed, and only regions that are yet to synapse retain the ability to form a crossover (Maguire, 1977).

This model would seem to be incompatible with interference in *Drosophila* and *C. elegans*, where SC appears to be complete before recombination is initiated (Dernburg et al., 1998; McKim et al., 1998). However, the SC is not a static structure. This was first recognized in the phenomenon of synaptic adjustment, wherein rearranged chromosomes that have different lengths initially appear to synapse based on homology, but then adjust so that the two chromosomes in each bivalent are equal lengths (reviewed in Moses et al., 1984). Studies in *C. elegans* found that crossovers locally alter SC, possibly to a form that is not permissive to additional crossovers (Libuda et al., 2013; Machovina et al., 2016). As discussed by Otto and Payseur (2019), these observations fit well with the proposal that interference propagates through the SC.

#### Counting Model

Like earlier models of interference based on a renewal process, Foss et al. (1993) proposed a model in which recombination intermediates that are randomly distributed along the chromosome can either become crossovers or non-crossovers, but intermediates are “counted” by a recombinase in such a way that two crossovers need to be separated by a certain number of non-crossovers. Although unable to explain interference data in *S*. *cerevisiae* (Foss and Stahl, 1995), a mathematic implementation of the counting model fit satisfactorily when tested against crossover data from other species and incorporated class II crossovers (Copenhaver et al., 2002; Housworth and Stahl, 2003; Stahl et al., 2004).

#### Beam-Film Model

The beam-film model of Kleckner et al. (2004) proposes that chromosomes behave like elastic beams covered on one face by a thin film that has flaws along its edges. When the chromosome is subjected to forces that cause the beam to expand, it does so to a greater degree than the film. This leads to the stretching of the film, which puts mechanical stress on it, resulting in the flaws cracking. When a crack is formed at one of the flaws, tensile stress in neighboring regions is relieved, with the release dissipating for some distance. Cracks may still occur at flaws on the film that are outside the reach of the stress relief perpetuating from the initial crack. This is applied to meiotic recombination by considering crossover precursors, such as DSBs or other intermediates, as the flaws on the film, with those that crack under mechanical stress being designated to become crossovers. Interference is explained by a crossover being able to prevent others in its vicinity by relieving the tensile stress on nearby flaws, consequently preventing them from cracking as well. A mathematical model based on beam-film can fit crossover distribution data from several species (Zhang et al., 2014a).

#### Compartmentalized Signaling Model

Studies in *C. elegans* suggested that the SC has liquid-like properties and Rog et al. (2017) proposed that this might help to explain crossover patterning. Zhang et al. (2018) showed that a set of four RING finger proteins, ZHP1-4, are part of a signaling network that functions within the SC to select early recombination intermediates for crossover designation. In *C. elegans*, there is always one crossover per pair of homologous chromosomes, so this signaling pathway designates only one crossover per compartment, with each SC being one compartment. Zhang et al. (2018) suggest that this may occur if both positive and negative activities within the SC to set up a wave of crossover designation potential.

#### Spatial Cluster Model

Fowler et al. (2018) proposed a model for crossover interference through DSB interference, in which DSB hotspots cluster within a chromosomal region of approximately 200 kb, with a limit of one DSB per cluster. This model fits the distance dependency of interference, as longer distances are more likely to span multiple clusters that act independently. However, this model seems to suggest that non-crossovers are also subject to interference, which does not appear to be the case (e.g., Miller et al., 2016). Additionally, since DSBs within each cluster are formed randomly, this model would suggest that crossovers forming at the boundaries of two neighboring clusters would not interfere. Another criticism of this model has been that it is based on studies in *S. pombe*, an organism in which interference had been reported to be absent (Mortimer and Fogel, 1974; Munz, 1994; Fowler et al., 2018). Fowler et al. (2018) provide evidence for weak crossover interference and suggest that interference in *S. pombe* occurs through clusters encompassing only one homolog instead of both, implying that clusters are distributed independently along each homolog, leading to efficient DSB interference without crossover interference.

### Mutations That Disrupt Interference

Genetic studies have identified many mutations that have an apparent effect on interference, but interpretation of these is often complicated. First, changes in interference would be expected to be inversely correlated with crossover number (e.g., weaker interference would be expected to increase crossover number), but changes in crossover number can occur without changes in interference. One important way this can happen is through loss of class I crossovers or increase in class II crossovers. Indeed, many mutations that appear to alter interference affect the absolute or relative numbers of class I and class II.

A good example is the *sgs1* mutant of *S. cerevisiae*. Oh et al. (2007) reported that crossover interference is reduced in *sgs1* mutants, which might be interpreted as evidence that Sgs1 plays a role in establishing interference; however, Zip2 foci, which mark sites designated to become class I crossovers, are normal in *sgs1* mutants, suggesting that normal interference is established (Fung et al., 2004). The solution to this apparent paradox came from subsequent studies that revealed that all crossovers in *sgs1* mutants require structure-selective endonucleases other than MutLγ (De Muyt et al., 2012; Zakharyevich et al., 2012). Thus, designation of crossover sites may occur normally, with interference, but maturation of these into crossovers is defective. Unlike MutLγ, other structure-selective endonucleases are not biased toward crossover resolution. This should result in a twofold decrease in class I crossovers. At the same time, loss of the anti-crossover activity of Sgs1 in the class II pathway results in an increase in those crossovers. Therefore, the reduction in interference reported by Oh et al. (2007) does not reveal a role for Sgs1 in the process of interference, but rather a change in ability to complete formation of class I crossovers combined with an increase in class II crossovers.

One protein that does appear to have a direct role in interference is Topoisomerase II in *S. cerevisiae.* Zhang et al. (2014b) analyzed distances between Zip3 foci in *top2* mutants and found that they were decreased relative to wild type, while total Zip3 foci were increased in the mutants. They hypothesized that topoisomerase II is required to relieve torsional stress at the site of crossovers. SUMOylation of Top2 by Ubc9 and subsequent ubiquitination by Slx5/8 is required for this function, and absence of Slx5 or Slx8 yields the same phenotype as in *top2* mutants. The sirtuin Sir2 physically interacts with Slx5/8 and activates its SUMO-targeted ubiquitin ligase (STUbL) activity to perform ubiquitination, and is also required for interference, as is Red1, another substrate of Ubc9.

## Crossover Assurance

### History

In most organisms, achiasmate chromosomes are rare (Hillers and Villeneuve, 2003). The first observation that all chromosomes exhibit at least one chiasma was made by Darlington and Dark (1932). They studied recombination in a grasshopper species with large variations in chromosome size. If crossovers were distributed randomly among the genome, smaller chromosomes would be less likely to experience at least one crossover, while larger chromosomes would be more likely to experience multiple crossovers. However, Darlington and Dark found that all chromosomes had at least one chiasma, indicating that there is a process that ensures at least one inter-homolog crossover on all chromosomes. Darlington hypothesized that chiasmata must be important for proper segregation of chromosomes (Darlington, 1937). Mather (1937) also graphed the number of chiasmata as a function of chromosome length and showed a minimum of one chiasma regardless of how short a chromosome is. He noted that the first chiasma is formed “irrespective of the length of the chromosome” and that additional chiasmata, if any, are subject to interference.

Another observation of assurance was made by Owen (1949). Callan and Montalenti (1947) studied the mosquito *Culex pipiens* and found what they interpreted as evidence for interference between intervals on different chromosome arms. This was unexpected, as interference between intervals separated by the centromere had not been found in any organisms prior. Owen reanalyzed their data and determined that interference need not cross the centromere in *Culex* if it is assumed that formation of a first crossover per bivalent is more favored than formation of subsequent crossovers. He defined this as a “primary chiasma” or “obligatory chiasma,” and reasoned that it is not on the same footing with other chiasmata, as it is required for synapsis and proper chromosome segregation. The reason behind the apparent cross-centromere interference in *Culex* was the short chromosome arms in this organism, resulting in bivalents that frequently had just one crossover, making it appear as if that crossover suppresses crossovers on the other arm. Owen’s interpretation held for an additional mosquito species studied by Callan and Montalenti, *Theobaldia longiareolata*, in which apparent cross-centromere interference was not observed due to its longer chromosome arms with a greater average number of chiasmata per bivalent. Jones (1984), noting that the distribution of chiasmata between chromosomes of similar sizes is more dispersed than would be expected if events were placed randomly, suggested that the lack of achiasmate chromosomes or univalents indicates that there must be one obligate chiasma per bivalent.

### The Obligate Crossover

The function of the crossover assurance mechanism is to ensure the generation of at least one crossover per bivalent. In recombination-dependent meiotic programs, a crossover is required between each pair of homologs to ensure a chiasma that will promote proper chromosome segregation at the end of meiosis I (Darlington, 1937; Hawley, 1988). A dramatic example of how crossovers promote segregation comes from studies of mammalian sex chromosomes. Koller and Darlington (1934) found that an obligate crossover was always formed between the *X* and *Y* chromosomes in rat. This conclusion was disputed for several decades due to the observation that in most mammals the *X* and *Y* associate end-to-end and do not exhibit visible chiasmata. However, Burgoyne (1982) later discovered via electron microscopy that a very distal chiasma forms between the *X* and *Y* to promote pairing. Burgoyne proposed an “*X–Y* crossover model” that suggests that there is a region of genetic homology between the *X* and *Y* chromosome (the “pseudoautosomal region”) in mammals that must experience an obligatory crossover during meiosis for proper chromosome segregation. All genes distal to the obligate crossover will be transmitted to both male and female offspring, behaving in pedigrees like autosomal genes.

The obligate crossover between the *X* and *Y* chromosomes must occur proximal to the sex determining region of the *Y* chromosome, *Sry*, which activates the male transcriptional program. Ashley (1994) used fluorescence *in situ* hybridization (FISH) to study how the *X* and *Y* chromosomes segregate into sperm, and determined that aneuploid products were infrequent, indicating that spermatocytes that experience *XY* non-disjunction fail to progress and differentiate into sperm. Hinch et al. (2014) later confirmed that PRDM9 binding sites, which coincide with recombination hotspots, are found in the human pseudoautosomal region between the *X* and *Y* chromosomes, *PAR1*. Intriguingly, recombination appears to drive sequence evolution more strongly in the pseudoautosomal region than on the autosomes. Recently, Papanikos et al. (2019) found that mouse ANKRD31 is essential to promoting double-strand break assurance, especially in the pseudoautosomal region. ANKRD31 deficiency leads to loss of an obligate crossover between the *X* and *Y* chromosome as well as consequent chromosome missegregation.

Exceptions to the requirement for an obligate crossover are seen in *Drosophila*, in which the X chromosomes do not experience a crossover in 10–15% of meioses and chromosome *4* never has crossovers. Nonetheless, these chromosomes segregate correctly in >99% of meioses due to an achiasmate segregation system (Hawley and Theurkauf, 1993), though the *X* still segregates with higher fidelity when it has at least one crossover (Koehler et al., 1996a).

### Mechanisms of Assurance

**FIGURE 4:**
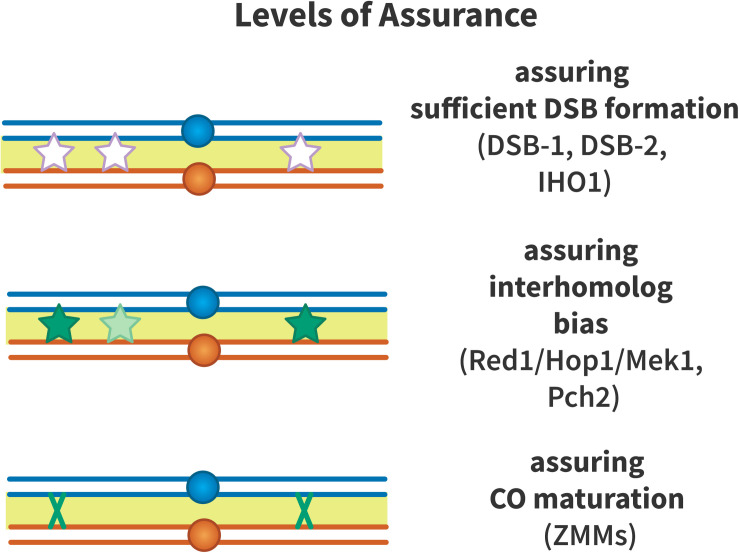
Levels of crossover assurance. Assurance is enforced at three levels. DSB-1 and DSB-2 form a checkpoint in *C. elegans* that ensures that sufficient double-strand breaks (DSBs, white stars) are formed to generate an obligate crossover. In mouse, IHO1 plays a similar role. From this pool of DSBs, inter-homolog recombination is promoted by Red1, Hop1, Mek1, and Pch2 to ensure that recombination intermediates (light green stars) engage with the homologous chromosome to form crossover precursors (dark green stars). The driving force to generate crossover precursors may be aggregation of ZMM proteins at recombination nodules or mechanical stress that must be relieved by crossover formation. Efficient crossover maturation (dark green X) is ensured by the ZMM proteins that define the class I crossover pathway.

Crossover assurance is enforced on multiple levels (Figure 4): assurance that sufficient DSBs are formed to generate an obligate crossover per chromosome, assurance of inter-homolog recombination bias, and assurance that a crossover is implemented by enforcing pro-crossover recombination pathways. These mechanisms are covered below.

#### Double-Strand Break Assurance

Meiotic DSBs are formed in excess relative to crossovers, likely to ensure that at least one crossover will be formed per homolog pair. A crossover assurance checkpoint involving the paralogs DSB-1 and DSB-2 was identified in *C. elegans* (Stamper et al., 2013). DSB-1 localizes to chromosomes during the time of DSB formation. When crossover formation is impaired, DSB-1 persists on chromosomes, suggesting that the time in which DSB formation is permitted is extended in *dsb-1* mutants. Failure to form an obligate crossover is sufficient for this phenotype to appear. DSB-2 similarly localizes to chromatin as DSBs form and disappears as RAD-51 foci appear, which mark early recombination intermediates. Association of DSB-2 with chromosomes is dependent on phosphorylation of the SUN domain protein SUN-1 and loading of RAD-51 at DSBs. Like DSB-1, DSB-2 persists on chromosomes when DSBs fail to form or recombination intermediates are not repaired (Rosu et al., 2013).

Double-strand breaks numbers are modulated by at least four different pathways in mouse (Dereli et al., 2021). DSBs regulate the number of SPO11-auxiliary protein complexes by activating the DNA damage response kinase ATM. DSBs additionally reduce IHO1 in their direct vicinity. IHO1 (homolog of yeast Mer2), a HORMAD1-interacting protein and SPO11 auxiliary protein, forms a chromatin-binding complex with MEI4 and REC114 that is required for DSB formation in mice (Dereli et al., 2021). In mice, DSB-dependent homologous recombination facilitates pairing and synapsis, which results in loss of IHO1, HORMAD1 and SPO11 complexes (Dereli et al., 2021). Lastly, DSBs along with the DNA damage response kinases ATM, ATR and PRKDC globally deplete IHO1 from chromosome axes. These pathways likely act to ensure that sufficient DSBs are generated to make an obligate crossover without creating a vast surplus of DSBs that threaten genomic integrity.

Double-strand break formation in yeast is regulated to ensure recombination on shorter chromosomes. The Spo11 accessory proteins Rec114 and Mer2 associate earlier and dissociate later from short chromosomes compared to longer chromosomes (Murakami et al., 2020). The mechanism that promotes this bias toward short chromosomes is unclear, but Rec114 and Mer2 accumulation are influenced by replication timing, while their dissociation is triggered by homolog pairing. ZMM-mutant budding yeast also generate higher numbers of DSBs than wild-type, and this control of DSB number is genetically separable from the pathway that connects DSB formation to meiotic progression (Thacker et al., 2014). These results suggest that homolog pairing, mediated by ZMM proteins, reduces DSB formation to prevent unproductive DSBs between paired homologs that are already engaging in inter-homolog recombination.

#### Inter-Homolog Strand Exchange Bias

An obligate crossover must occur between homologous chromosomes to ensure proper chromosome segregation. Mechanisms that promote inter-homolog recombination over recombination between sister chromatids have been described. In yeast, Hop1 and Red1 function structurally in axial/lateral elements and function jointly with Mek1 to enforce a barrier to inter-sister recombination. The chromosome axis protein Hop1 is phosphorylated in response to DSBs, and then triggers dimerization of the meiosis-specific kinase Mek1, which phosphorylates proteins that limit inter-sister recombination (Niu et al., 2005, 2007). One target of Mek1 is Hed1, a meiosis-specific protein that binds Rad51 and suppresses its activity (Tsubouchi and Roeder, 2006; Busygina et al., 2008). Mek1 also phosphorylates the Rad51 binding partner Rad54, reducing Rad51’s activity (Niu et al., 2009). In organisms that use the meiosis-specific strand exchange protein Dmc1, Dmc1 is required for interhomolog bias (Brown et al., 2015), and thus Rad51 activity is inhibited to allow Dmc1 to function as the major meiotic strand exchange protein (Callender et al., 2016).

Pch2 is a highly conserved ATPase that has been implicated in many processes in meiosis. In yeast, Pch2 has been found to control association between Zip3, which localizes to crossover precursors, and the chromosome axis proteins Hop1/Red1. Although Pch2 is not required to generate crossovers at normal levels, *pch2* mutants appear to have reduced interference and lack crossover assurance (Chakraborty et al., 2017). Joshi et al. (2009) suggested that Pch2 remodels the chromosome axis into an array of crossover control modules that interact over a long range to ensure that there is an obligate chiasma and that crossovers are maximally spaced. *C. elegans pch-2* mutants lose access to recombination with the homologous chromosome early, suggesting that PCH-2 is required to maintain interhomolog recombination bias (Deshong et al., 2014).

#### Robust Crossover Designation and Maturation

Shinohara et al. (2008) studied null mutants of the ZMM gene *SPO16* and found that, while crossovers are reduced in these mutants, the residual crossovers that are observed display interference. Furthermore, Spo16 interacts with Zip4 via co-immunoprecipitation, and assembly of Spo16 foci depends on Zip1 and Zip3, but not on Msh4. Shinohara et al. proposed that the ZMMs may consist of two sub-assemblies: one that enforces interference by stabilizing crossover precursors at the DNA level, and one that promotes crossovers at these sites to generate assurance.

In mouse, the E3 ligases RNF212 and HEI10 generate crossovers by SUMOylating recombination proteins (Reynolds et al., 2013; Qiao et al., 2014). These factors are thought to act as a checkpoint that pauses recombination by altering the turnover time of meiotic recombination proteins such as DMC1 and MutSγ. This may stall intermediates at an early step in recombination to ensure that the appropriate pro-crossover factors are present to establish crossover designation (Prasada Rao et al., 2017).

In *C. elegans*, DSBs are preferentially repaired as COs in the absence of inhibitory effects from other recombination precursors (Rosu et al., 2011). Rosu et al. (2011) used excision of the *Mos1* transposon to make a targeted DSB in *spo-11* mutants, in which no meiotic breaks are formed. They found that nearly all interhomolog repair events in these mutants were COs.

Cytoskeletal forces have also been shown to promote synapsis, recombination, and crossover assurance through signaling via SUN- and KASH-domain proteins. In *Arabidopsis*, the kinesin AtPSS1 is required for crossover assurance (Duroc et al., 2014). In *Atpss1* mutants, some chromosomes lack an obligate crossover, while the number of total meiotic crossovers is comparable to wild type. This kinesin directly interacts with the KASH-domain proteins WIP1 and WIP2. Ndj1/Tam1, a protein that tethers telomeres to the nuclear envelope, stabilizes interactions between homologous chromosomes and thus may promote inter-homolog recombination (Conrad et al., 1997; Chua and Roeder, 1997).

In addition to crossover designation, it is important to ensure effective crossover maturation to generate the obligate crossover. Wang et al. (2017) modeled human oogenesis using the beam-film framework and found that a lower crossover maturation efficiency could explain the higher number of achiasmate bivalents in oocytes.

## Crossover Suppression

### History

The final patterning event we will discuss is crossover suppression associated with specific chromosomal features. Most notably, suppression occurs both in the pericentromeric region and telomeric regions. Centromeric suppression is thought to be crucial for proper chromosome segregation in humans, budding yeast, fission yeast, and *D. melanogaster* (Koehler et al., 1996a; Lamb et al., 1996; Rockmill et al., 2006; Smith and Nambiar, 2020). Two models have been proposed to explain how proximal crossovers promote non-disjunction (Orr-Weaver, 1996). First, proximal crossovers may disrupt sister-chromatid cohesion at the centromere. Since proximal cohesion needs to be maintained until separation of sisters at anaphase II, disruption may lead to premature separation of sister chromatids (PSSC). The second model is the converse: Because proximal cohesion is not released at anaphase I, a proximal crossover causes the homologs to become entangled, resulting in both segregating to the same daughter. One or both suggestions may account for the existence of mechanisms that reduce or eliminate centromere-proximal crossovers. It may be that disruption of cohesion and PSSC is more important in organisms with point centromeres and relatively small regions of pericentromeric cohesion, but entanglement is more of a risk in organisms in which centromeres are embedded in large blocks of heterochromatin are more expansive pericentromeric cohesion.

The first observation of centromere-proximal crossover suppression, now known as the centromere effect, was made by Beadle (1932), when he observed a decrease in crossing over in medial regions of a *Drosophila* chromosome when they were moved closer to the centromere in a translocation stock. Beadle studied a translocation that attached the right half of *3R* to the centromere (or “spindle fiber” as he called it) of chromosome *4.* In homozygous translocation flies, the percent crossing over in the intervals now closest to the *4* centromere dropped dramatically, while crossing over within more distant intervals was not affected. Beadle concluded that the decrease in crossovers in these two regions was a result of them having been moved closer to the chromosome *4* centromere.

In a 1936 review, Mather presented more evidence for the idea that the centromere exerts control over the distribution of crossing over (Mather, 1936). He used recombination data from other researchers to show that while genetic loci are well spaced out in the middle of the chromosome (as in the case of the telocentric *X*) or chromosome arm (as in the case of metacentric *2* and *3*), a clear crowding of loci can be seen around the centromere. This is indicative of low recombination frequency near the centromere, which led Mather to speculate that crossing over in a particular region is a function of its distance from the centromere.

### Factors Influencing the Centromere Effect

#### Temperature

Harold Plough was the first to study the effects of environmental changes on crossing over (Plough, 1917a). His data showed that while starvation and food type did not affect crossover frequencies in *Drosophila*, temperature did. When studying the regions between chromosome *2* markers close to and flanking the centromere, he observed an increase in crossover percentages at both higher (31 C) and lower (13 C) temperatures, which he attributed to a physical change that was occurring in chromatin structure. This increase in centromere-proximal crossover frequencies at high temperatures was later shown to hold for chromosomes *1* and *3* as well (Plough, 1921; Stern, 1926). Mather (1939) showed that heterochromatin was responsible for crossing over being highly sensitive to temperature, and speculated that variations caused by other environmental factors could also be a result of their effect on heterochromatin. A more recent study also found that increasing temperature leads to an increase in proximal crossovers in barley, a species in which crossovers are primarily distal under typical temperature conditions (Phillips et al., 2015).

#### Maternal Age

Bridges (1915) showed that maternal age can influence crossing over in *Drosophila* when he observed that older females showed a decrease in pericentromeric crossover percentages on chromosome *2*. Later, he showed that this decrease was most drastic in the center of metacentric chromosome *3*, decreasing as loci further from the centromere were considered (Bridges, 1927). Thus, Bridges showed in 1915, nearly two decades before Beadle’s data on suppression of crossing over near the centromere, that crossover percentages between centromere-proximal markers were as low as 5%. Although it would have been impossible for Bridges to have noticed this centromere-proximal reduction in crossing over as he had no knowledge of the physical distances between these markers, this data is possibly the first evidence of the centromere effect.

#### Heterochromatin

Mather (1939) studied how both the centromere and heterochromatin influenced crossing over in the *Drosophila X* chromosome using various inversion stocks in which markers that were ordinarily centromere proximal were moved to more distal positions and *vice versa*. His results led him to confirm his earlier belief that crossover frequencies in euchromatic regions are dependent on their distance from the centromere. Mather also observed some amount of crossing over in heterochromatic regions that had been moved farther away from the centromere and speculated that these levels may also be dependent on distance from the centromere. Baker (1958) did not agree with these findings, and thought that the crossovers Mather reported were most likely in adjacent euchromatin since the markers he’d used did not delineate heterochromatin very precisely. Baker supported this claim by showing virtually no crossing over in a *Drosophila virilis* stock in which a large chunk of chromosome *5* heterochromatin had been moved to the tip of chromosome *3* via reciprocal translocation. Since then, it has been widely believed that crossing over in heterochromatic regions is uncommon, if not absent (Carpenter and Baker, 1982; Szauter, 1984). It was also thought that pericentromeric heterochromatin was causing crossover suppression near the centromere, possibly by restricting access to recombination proteins (Westphal and Reuter, 2002), as structural differences in the synaptonemal complex are seen in the two types of chromatin (Carpenter, 1975; Stack, 1984).

Westphal and Reuter (2002), investigated the role of chromatin in centromeric crossover suppression by studying the effects of dominant suppressor-of-variegation mutations on crossover frequencies. These mutations are thought to cause changes in heterochromatin structure, consequently leading to suppression of heterochromatin-mediated gene silencing. The authors hypothesized that this may result in a change in crossover patterning in heterochromatin as well as flanking euchromatin. In support of this hypothesis, of the 46 mutations they tested, 16 increased crossing over in pericentromeric heterochromatin. A 2012 study in *A. thaliana* also showed the same dependence of crossover frequency on chromatin states, when a DNA hypomethylated mutant that showed pericentromeric transcriptional activity also showed an increase in centromere proximal crossovers (Yelina et al., 2012). However, this study, along with others that looked at hypomethylated *Arabidopsis* mutants, observed no change in crossover suppression in pericentromeric regions (Colomé-Tatché et al., 2012; Melamed-Bessudo and Levy, 2012; Mirouze et al., 2012). This was a surprising result as pericentric heterochromatin is hypermethylated in wild-type plants and would have been expected to show increased rates of recombination under conditions of DNA methylation loss. The authors of these studies propose that lowering DNA methylation levels may be making chromatin that is already open further accessible to crossover formation.

Despite considerable evidence that heterochromatin plays an important role in suppressing centromere-proximal crossovers, there is also support for the hypothesis that distance from the centromere is just as, if not more, critical. Mather (1939) showed in *Drosophila* that a euchromatic interval moved farther from the centromere but closer to a larger length of heterochromatin showed less crossover suppression than an interval moved closer to the centromere but near a smaller length of heterochromatin. He concluded from this that the centromere fiber effect is more a consequence of centric action than proximity to heterochromatin. Lindsley and Sandler (1977) showed that despite having the largest amount of pericentromeric heterochromatin, the *Drosophila X* chromosome had significantly higher crossing over in proximal euchromatin than either autosome. Their data also suggested that the centromere effects of each chromosome in *Drosophila* remained roughly the same despite them having different amounts of heterochromatin. This has been corroborated by Yamamoto and Miklos (1978), who also showed that crossing over in the proximal euchromatin of *Drosophila X* chromosomes with varying extents of heterochromatin deletions depended more on the distance from the centromere than on amount of heterochromatin. They suggested that heterochromatin acts only as a passive spacer between the centromere and euchromatin, implying that chromosome structure and mechanics, as well as DNA content, would be crucial in defining the centromere’s control over crossing over in an organism. However, they decidedly acknowledge that when combined with other data on recombination being genetically controlled (Catcheside, 1977; Lindsley and Sandler, 1977), crossover suppression near the centromere appears to be mediated by a complex system with multiple facets of control.

More recently, Hartmann et al. (2019) observed that when a large block of heterochromatin was inserted into chromosome *2R* of *Drosophila*, there was no significant crossover suppression in adjacent intervals, suggesting that the centromere effect does not arise from an innate property of heterochromatin. They also went on to show that pericentromeric crossover suppression seems to be through two mechanisms: complete suppression in the densely staining, highly repetitive alpha heterochromatin, and the centromere effect that exhibits a distance-dependent suppression that extends far into euchromatic sequences. This is consistent with Yamamoto and Miklos’ conclusion that suppression of crossovers in the vicinity of the centromere has multiple causes.

#### Synaptonemal Complex

Carpenter (1975) observed using electron microscopy that the structure and morphology of the SC is different in the pericentromeric heterochromatin as compared to more distal regions on the chromosome that are euchromatic. This was further confirmed by her observation that the SC of the largely heterochromatic chromosome *4* in *Drosophila* usually had only heterochromatic morphology. Combined with her observation that all recombination nodules in her experiment were found in distal chromosomal regions that had euchromatic SC morphology, this raises the possibility that the SC could be mediating the centromere effect.

Stack conducted an electron microscopic study of meiotic chromosomes from mice and two angiospermous plants and showed that the SC is shorter and more under-represented in the heterochromatic regions of meiotic chromosomes, suggesting a reason for the lack of crossing over in heterochromatin (Stack, 1984). In addition to confirming Carpenter’s observations of SC structure being different in euchromatin versus heterochromatin, he also showed that heterochromatic SC is more densely enclosed in compact chromatin, which he implied could sterically inhibit essential recombination enzymes from accessing it.

### Mechanistic Insights Into the Centromere Effect

Although much is known about various factors influencing centromere-proximal crossover suppression, the exact mechanism behind the centromere effect is still unknown. One hypothesis is that DSB formation is suppressed near the centromere. This is supported by a 2006 study in *Drosophila* that showed that the DSB cytological marker γ-His2Av failed to colocalize with HP1, a marker for heterochromatin (Mehrotra and McKim, 2006). As colocalization was observed in irradiated flies, DSBs were believed to be excluded from heterochromatin in wild-type meiotic cells. Further support for this proposal comes from a study in *S. pombe* showing that the pericentric cohesin complex actively represses pericentric DSB formation (Nambiar and Smith, 2016) (Figure 4). The mechanism of action proposed involves the heterochromatin protein Swi6 recruiting Rec8-Psc3 – mitotic cohesin complex subunits – to pericentric regions. Both Swi6 and Psc3 function to keep out Rec11, a meiotic cohesin complex protein, and prevent it from binding Rec8. This ensures that Rec11 is unable to recruit linear element protein Rec10, leading to the protein responsible for creating DSBs, Spo11, remaining inactive. This leads to the suppression of DSB formation and therefore meiotic recombination. Furthermore, the authors suggest that a molecular mechanism involving different cohesin complexes acting at pericentromeric heterochromatic regions to suppress recombination is a mechanism conserved across those species in which heterochromatin is found at the pericentromere.

**FIGURE 5:**
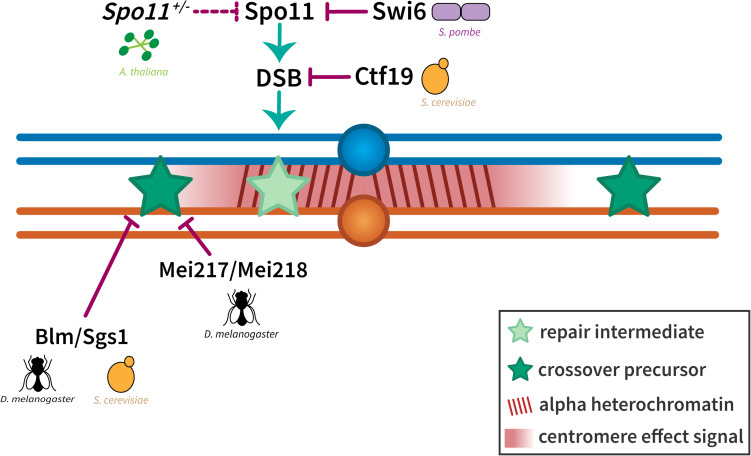
Suppression of crossovers near the centromere. The exclusion of crossovers near the centromere seems to be enforced at two levels. Crossover suppression in the highly repetitive alpha heterochromatin has been shown to be a result of the prevention of DSBs either through local chromatin organization (in *S. cerevisiae*) or the inactivation of Spo11 (in *S. pombe* and *A. thaliana*). Crossover suppression in the less repetitive beta heterochromatin and the proximal euchromatin has been shown to be regulated through Bloom syndrome helicase/Sgs1 (in *D. melanogaster* and *S. cerevisiae*) and dependent on distance from the centromere (represented by a centromere effect signal shown in red). Homologous chromosomes are shown in blue and orange with dark red lines in the pericentromeric region representing alpha heterochromatin. Light and dark green stars represent repair intermediates and crossover precursors, respectively.

Spo11 itself has been observed to play a role in crossover patterning, when a 2018 study in *Arabidopsis* determined that a Spo11 hypomorph that showed a proportional reduction in the number of DSBs along the chromosome was able to disproportionately decrease the number of crossovers in pericentromeric regions (Xue et al., 2018) (Figure 5). However, studies in budding yeast that mapped DSBs using whole-genome methods and ssDNA accumulation have shown that although DSBs are suppressed near the centromere, the length of chromosome along which suppression is observed, originally thought to be 20 kb (Gerton et al., 2000), and later 8–10 kb (Buhler et al., 2007), is really only 1–3 kb (Pan et al., 2011). Considerable DSB activity was seen in pericentromeric regions, including hotspots within 10 kb of the centromere (Blitzblau et al., 2007). Further support for the presence of DSBs in pericentromeric regions comes from Symington and Petes (1988), who showed, using strains that were heterozygous for restriction sites within the centromeric sequence, that there are widespread gene conversion (GC) events occurring at centromeric regions in yeast. Symington and Petes (1988) concluded that in budding yeast, the rates of centromeric gene conversion events during meiosis are similar to non-centromeric regions, and since GC events, while leading only to non-crossovers, still arise from DSB repair, these results show that DSBs are indeed formed in centromeric regions. More recently, a 2015 study threw some light on the molecular mechanism behind pericentromeric crossover suppression in budding yeast by showing that the kinetochore Ctf19 complex is important in two ways: It prevents DSB formation within a 6 kb region of the centromere and also suppresses inter-homolog recombination within the pericentromere (Vincenten et al., 2015). The authors suggest that prevention of DSB formation is through regulating recruitment of chromosomal axis proteins/local chromatin organization, and that crossover suppression is through recruitment of cohesins at pericentromeric regions, which they speculate promotes inter-sister repair over inter-homolog recombination. These cohesins were proposed by Vincenten et al. (2015) to recruit Zip1, an SC protein that has been implicated in promoting pericentromeric inter-sister repair at the expense of inter-homolog repair (Chen et al., 2008).

Similar results were later also seen in *Drosophila* when Comeron et al. (2012) detected gene conversion in regions of low or no crossing over, and showed that non-crossover recombination events are more uniformly distributed along the genome than crossovers were. In a subsequent study, Miller et al. (2016) found that non-crossover gene conversion events are not sensitive to the centromere effect. Both studies were able to evaluate non-crossover repair products by detecting changes in single nucleotide polymorphisms in unique-sequence centromere-proximal regions.

Sgs1, the budding yeast ortholog of Blm helicase, was shown by Rockmill et al. (2006) to be an important factor in preventing precocious separation of sister chromatids. Their study showed that the most common cause of spore inviability in budding yeast is aneuploidy arising from PSSC, which is often associated with, and promoted by, crossing over in centromere-proximal regions, further supporting the suggestion that proximal crossovers lead to non-disjunction.

In the experiments of Brand et al. (2018) described above, in which *Drosophila mauritiana* Mei-217 and Mei-218 were put into *D. melanogaster*, the researchers noted that crossovers were especially elevated in telomere- and centromere-proximal regions. Furthermore, they saw an increase in crossovers in the interval that spans the centromere in chromosome *2*, leading them to conclude that the Mei-217 and Mei-218 proteins may be involved in centromeric and telomeric crossover suppression.

It is also possible that the mechanisms behind the centromere effect are primarily structural, and that the number of centromeres is able to influence the strength of the effect. In support of this argument, Redfield showed in studies of triploid crossing over that there was a 1.5- and 3-fold increase in crossover frequencies at the center of chromosomes *2* and *3 –* where the centromere is located - when compared to diploid females (Redfield, 1930, 1932). This increase in crossover frequencies decreased with distance from the centromere, which suggests that an increased total number of centromeres could be acting as a molecular sponge and “soaking up” a CE signal, ultimately leading to a weaker centromere effect.

While some genes involved in facilitating the centromere effect have come to light in the past few years, there is much that is still unknown. It will be important to look closely at whether the mechanisms behind the centromere effect are genetic or structural, how this effect relates to other patterning events, and how crossover suppression in centromeric regions is different from suppression in telomeric regions.

## Interplay Between Patterning Phenomena

Recent work has suggested that crossover assurance, interference, and suppression may be interconnected or part of an overarching crossover control mechanism. Interactions between crossover patterning phenomena are highlighted below.

### Interference and the Centromere

The earliest suggestion that the centromere can impede interference comes from Muller’s 1916 paper where he speculated that coincidence values on the second chromosome would be different if crossovers on the same chromosomal arm are compared to crossovers on either side of the centromere even when separated by the same distance in both cases (Muller, 1916a). He suggested that this could be due to chromosomes bending in the middle, or due to differences in structure at centromeric regions. In support of Muller’s finding, Kikkawa (1932) showed an increase in coincidence values in the central regions of the chromosome, which led him to conclude that crossover interference in the sections spanning the centromere is weaker than elsewhere on the chromosome. Graubard (1934) also showed that crossovers on one chromosomal arm do not interfere with crossovers on the other. These findings imply that the centromere can block an interference ‘signal.’ However, this block does not seem to be complete, as Graubard also observed that coincidence values between two small, adjacent intervals on either side of the centromere do not equal one (as they should for zero interference) but display less interference than intervals of the same size that are located on the same chromosomal arm. He concluded that coincidence values in a particular interval are not affected by the presence of either a terminal or central centromere but instead depend on the length of the interval being considered.

These conclusions from studies in insects were extended to *Neurospora* by Bole-Gowda et al. (1962), who demonstrated through tetrad analysis that there is little to no interference between crossovers on either side of the centromere. However, more than 30 years later, Colombo and Jones (1997) showed using previously published statistical analyses of chiasma data from grasshoppers that interference seemed to be able to act across the centromere when coincidence values were calculated, or distances from the nearest chiasma and the centromere were correlated. This led them to conclude that interference is blind to the presence of a centromere. Later, Kaback et al. (1999) also showed that in *S. cerevisiae* recombination on one chromosome arm is affected by changes in the size of the other. They concluded that this is indicative of the interference ‘signal’ having the ability to pass through the centromere, since their results suggest that crossover interference in budding yeast increases with the size of the chromosome. They explained their stance by pointing out that the model in which the centromere blocks interference ignores the fact that centromeric markers have greater physical separation and is based only on genetic mapping data. They proposed that in centromere-proximal regions, particularly in organisms that have large amounts of pericentromeric heterochromatin, interference appears to be blocked by the centromere because it has a much greater distance to cover. However, this does not seem to be the case across species, as Fowler et al., who in 2018 proposed the DSB hotspot clustering model of interference in *S. pombe*, speculated that the reason an interference ‘signal’ is unable to travel across the centromere in certain organisms is due to DSB hotspot clusters not being formed in pericentromeric regions (Fowler et al., 2018). Further, since *Drosophila* and other metazoa differ greatly from yeast in their chromatin structure, sequence, and even centromeric SC morphology, the role that the centromere plays in relaying or blocking an interference signal may well be different across these species.

In a 2012 study in *A. thaliana*, Yelina et al. (2012) showed increased centromere-proximal crossovers in a *methyltransferase1* (*met1*) mutant with hypomethylated DNA. However, they observed no difference when the total number of crossovers in *met1* mutants was compared to wild type, which they took as an indication that crossover interference and crossover homeostasis were acting to remodel the changed crossover landscape in the hypomethylated mutant to compensate for the increased centromere-proximal crossovers. Similarly, an *Arabidopsis* study by Xue et al. (2018) showed that pericentromeric crossover frequencies fell in *SPO-11* hypomorphs, where DSB numbers are drastically reduced. The authors suggest that this could allow medial crossover frequencies to rise, which in turn could inhibit pericentromeric crossovers through interference.

Hatkevich et al. (2017) showed that *D. melanogaster* mutants deficient for Bloom syndrome helicase lost crossover interference and crossover suppression in centromeric regions. They went on to speculate that interference and the centromere effect may be interdependent, with the centromere effect reinforcing interference by ensuring that most crossovers happen medially along the chromosome. However, they also state that this could be unlikely as the *D*. *melanogaster* telomere effect, or the suppression of crossovers in the telomeric regions, is not as strong as it should be for the two crossover suppression events to be coordinately reinforcing interference. The experiments of Brady et al. (2018) discussed above also provide a link between crossover suppression in pericentromeric regions and interference. However, Brady et al. (2018) found that flies mutant for *mei-41*, which encodes the *Drosophila* ortholog of ATR kinase, lose class I crossovers but appear to retain suppression of centromere-proximal crossovers. The authors proposed that crossover suppression is established earlier than other patterning processes.

These results suggest that it is possible for the centromere effect and interference to be independent events operating through entirely different mechanisms that happen to require some of the same factors to proceed correctly. However, it remains possible that the two events are established through the same mechanism, involving a crossover suppression signal traveling outward from the centromere/existing crossovers, mediated by some common proteins but separated temporally during meiosis.

### Interference and Assurance

As pointed out by Wang et al. (2015), the need for crossover assurance is obvious, since at least one chiasma is necessary to promote accurate segregation of homologs, but whether there is a selective advantage of having interference is less clear. Several possible functions for interference have been suggested. It has been suggested that crossovers near one another may lead to a lack of sufficient cohesion to stabilize the bivalent (Fowler et al., 2018), but this should only be the case for two-chromatid DCOs. A study in *C*. *elegans* found that interference plays a key role in ensuring proper chromosome segregation by limiting the number of crossovers per homolog (Hollis et al., 2020); however, this may be a problem unique to organisms without defined centromeres. Interference may serve to limit the number of crossovers, but in Arabidopsis, increasing crossovers by three–sixfold has no apparent negative effect on fertility or chromosome segregation (Crismani et al., 2012; Girard et al., 2015). Wang et al. (2015) proposed that spacing out crossovers strikes a balance between the advantages of generating new combinations of alleles and the disadvantages of breaking up co-adapted combinations.

Several observations suggest that the strength of interference can be subject to selection. Brand et al. (2018) found that replacing *D. melanogaster* Mei-217 and Mei-218 with the *D. mauritiana* orthologs captured many of the crossover differences between these species, including reduced interference. They also reported strong evidence for positive selection within *mei-218* in both species, suggesting this might be associated with changes in interference and therefore in crossover numbers. Gorlov and Gorlova (2001) simulated a cost-benefit analysis of recombination and suggested that natural selection balances, through changes in interference, the positive and negative effects of recombination. The selective pressures that might drive higher or lower crossover rates are unknown.

There are also special cases where selection may operate on the strength of interference. In *C. elegans*, chromosomes are holocentric in somatic cells, but in meiosis the crossover produces an asymmetry that results in one end being selected for assembly of the kinetochore (Albertson and Thomson, 1993; Martinez-Perez et al., 2008). Two crossovers on the same bivalent may disrupt this process, leading to selection for absolute interference (one crossover per bivalent). Another possible case is suggested by studies of autopolyploids that arise from intraspecies whole-genome duplication. In early stages of allopolyploidy, homologous chromosomes form quadrivalents, and multiple chiasmata can lead to missegregation (Yant et al., 2013). Bomblies et al. (2016) proposed that increases to the strength of interference might promote more accurate meiotic segregation by reducing the number of chiasmata.

Another function for interference might be to promote assurance. The phenomenon of crossover homeostasis might support a dependency relationship between assurance and interference. Martini et al. (2006) studied budding yeast mutants with hypomorphic alleles of *spo11* and found that reduced DSB levels did not lead to the same reductions in crossover levels, suggesting an assurance mechanism that is buffered to some degree against different numbers of precursors. Homeostasis might involve a mechanism to produce a set number of crossovers per meiosis, within some range. This could explain the interchromosomal effect in *Drosophila*, where the presence of structural rearrangements on one chromosome both prevent it from crossing over with its homolog and lead to increased crossovers on other chromosomes (Lucchesi and Suzuki, 1968; Crown et al., 2018). If a set number of crossovers must be achieved, interference would limit the number of crossovers on larger chromosomes, forcing smaller chromosomes to cross over.

Finally, it is possible that interference and homeostasis are merely byproducts of the process through which a precursor is designated to become a crossover. In the beam-film model, mechanical stress provides a driving force to generate designation of an obligate crossover, and the resulting distance-dependent release of stress leads to both spacing of crossovers (interference) and homeostasis (Wang et al., 2015). The compartmentalized signaling model proposed by Zhang et al. (2018) is similar in this regard.

## Conclusion

Although they have been traditionally defined as distinct phenomena, there is a great deal of overlap between the proteins and potentially the mechanisms underlying crossover interference, assurance, and the centromere effect. It is possible that a common mechanism is responsible for all three phenomena, whether that is aggregation of pro-crossover factors, mechanical stress, or another driving force that limits crossovers to specific regions of the genome. It is also apparent that the currently known and proposed mechanisms for crossover patterning operate at a wide range of scales, from the DNA level to the chromosome and inter-chromosome levels. Future work may focus on illuminating the connection between these mechanisms to better understand the overarching regulation of crossover patterning.

## Author Contributions

All authors listed have made a substantial, direct and intellectual contribution to the work, and approved it for publication.

## Conflict of Interest

The authors declare that the research was conducted in the absence of any commercial or financial relationships that could be construed as a potential conflict of interest.
